# Formic Acid Pretreatment Enhances Untargeted Serum and Plasma Metabolomics

**DOI:** 10.1021/acs.analchem.5c03725

**Published:** 2025-10-07

**Authors:** Tereza Kacerova, Elisabete Pires, Abigail Dixon, Rachel Williams, Isabelle Legge, Mia Hippisley, Abi G. Yates, A. David Smith, Daniel C. Anthony, Fay Probert, James S. O. McCullagh

**Affiliations:** † Chemistry Research Laboratory, Department of Chemistry, 6396 University of Oxford, Oxford OX1 3TA, U.K.; ‡ Department of Pharmacology, University of Oxford, Oxford OX1 3QT, U.K.

## Abstract

Untargeted metabolic profiling of plasma and serum by liquid chromatography–mass spectrometry (LC-MS) is becoming increasingly important in clinical and translational research; however, sample preparation protocols can have a significant impact on study outcomes, and there is currently a lack of standardized approaches. In this study we demonstrate that pretreatment of serum and plasma samples with 1% formic acid (FA, v/v) prior to acetonitrile (MeCN)-induced protein precipitation significantly enhances analytical performance in untargeted metabolomics using reversed-phase liquid chromatography (RPLC)-MS. We show an increase in sample preparation reproducibility and signal intensity across both positive and negative ionization modes. In two independent serum cohorts (OPTIMA and VITACOG), FA-based extraction improved multivariate modeling (orthogonal partial least-squares discriminant analysis, OPLS-DA), with consistently higher classification accuracy, sensitivity, and specificity, alongside reduced variability and increased fold-changes in discriminatory compound-features. We investigated factors potentially involved in the enhanced performance and observed outcomes consistent with the disruption of noncovalent protein–metabolite interactions and the stabilization of labile species. We found no correlation with either protein depletion or differential adduct formation. The results were also not attributable to lowering pH after metabolite extraction. In summary, we demonstrate that FA pretreatment of plasma and serum, prior to protein precipitation, significantly improves sample reproducibility and detection sensitivity in untargeted RPLC-MS metabolomics. This optimized sample preparation strategy offers clear advantages for clinical and translational metabolomics, with the potential to enhance biomarker discovery and metabolic phenotyping.

## Graphical abstract

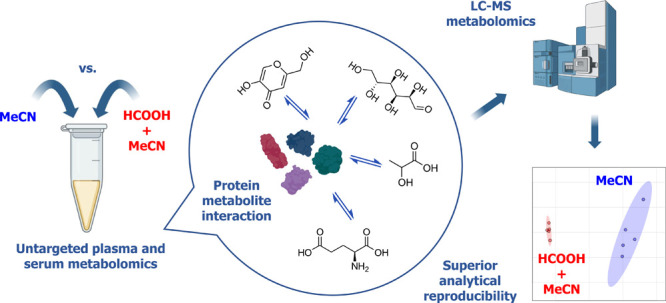

## Introduction

Liquid chromatography–mass spectrometry (LC-MS)-based metabolomics is increasingly used for biomarker discovery, disease stratification, and personalized medicine applications.^1–3^ However, clinical studies are often challenged by substantial interindividual biological variability, while disease-associated metabolic alterations are typically subtle, particularly in early or prodromal stages.^3–5^ In this context, technical variation introduced during sample preparation can significantly compromise analytical sensitivity and reproducibility.^6^ Optimization and standardization of preanalytical workflows is therefore essential to maximize metabolome coverage, reproducibility and ensure optimal study results.

Protein precipitation using acetonitrile (MeCN) is the standard approach for preparing plasma and serum samples for LC-MS.^4,6–8^ Although effective at protein removal, this approach is known to introduce variability in the metabolite recovery profile, especially for low-abundance or protein-bound small molecules.^9,10^ This may be particularly relevant for circulating metabolites that exhibit partial or reversible binding to plasma proteins *in vivo*. These include lipophilic compounds, steroids, and organic acids, as their binding may impair extraction efficiency and reduce quantification precision.^11^ Acid-assisted metabolite extraction protocols using low concentrations of formic acid (FA ≤ 0.3%) have been shown to improve stability in targeted assays.^12,13^ However, the systematic evaluation of FA-assisted extraction for untargeted metabolomics, including optimal concentration ranges and analytical impact, has not been previously reported. To address this, we systematically evaluated the pretreatment of serum and plasma samples with varying concentrations of FA (0.3–5% by volume) and found that 1% FA, when applied prior to MeCN precipitation, significantly enhanced metabolite recovery. This was reflected by increased feature detection and improved reproducibility in reversed-phase LC-MS (RPLC-MS) under both positive (pos.) and negative (neg.) ionization modes. We further examined potential mechanisms underlying these improvements, including disruption of protein–metabolite interactions, pH modulation, and alterations in ionization efficiency. Finally, we performed comparative analyses in two clinical cohorts (OPTIMA and VITACOG),^14,15^ studies designed to investigate cognitive decline, and demonstrated that FA-treated samples consistently yielded superior multivariate model performance, greater fold change (FC) in discriminatory compound-features, and reduced technical variability. Collectively, our findings suggest that FA pretreatment constitutes a simple and effective optimization of metabolite extraction from serum and plasma samples with the potential to benefit untargeted LC-MS metabolomics workflows in clinical research.

## Experimental Section

### Sample Preparation and LC-MS Analysis

Plasma and serum metabolites were extracted using MeCN with or without pretreatment with formic acid (1%, final concentration). Samples were vortexed and centrifuged, and supernatants were collected for LC-MS analysis (Supporting Information, eMethods 1.1–1.2). RPLC-MS was performed on a Xevo G2-XS QTOF mass spectrometer coupled to an Acquity UPLC system under gradient elution (Waters Limited, Wilmslow, UK). Data processing was performed using Progenesis QI and in-house R scripts and MetaboAnalyst 6.0.^16^ For detailed method description, including RPLC-MS (pos. and neg. ionization modes), hydrophilic interaction liquid chromatography MS (HILIC-MS), anion-exchange chromatography (AEC-MS), and MALDI-TOF MS analyses, as well as complete experimental protocols, see the Supporting Information, eMethods 1.3–1.6.^17^

### Statistical Analysis

Untargeted clinical LC-MS data were analyzed using R software (v4.2.1) with in-house scripts (Supporting Information, eMethod 1.7). Multivariate analysis was performed using orthogonal partial least-squares discriminant analysis (OPLS-DA) with 10-fold cross-validation, repeated permutation testing, and variable importance in projection (VIP) scoring, as previously described (Supporting Information, eMethod 1.8).^18,19^ Full methodological details, statistical workflows, and clinical information for the OPTIMA and VITACOG cohorts are provided in the Supporting Information, eMethods 2.1–2.3. Univariate analysis involved fold change (FC) and *t* test *p*-value calculations between extraction methods, visualized as volcano plots (FC > 2, *p* < 0.05). To maximize sensitivity, multiple testing corrections were not applied unless stated. Principal component analysis (PCA) assessed group variation. Remaining analyses were conducted in GraphPad Prism 9 (significance *p* < 0.05).

## Results

### Formic Acid Pretreatment Enhances Sensitivity and Reproducibility in Metabolic Profiling

Protein precipitation with MeCN is widely used in LC-MS-based metabolomics to remove residual protein, simplify the sample matrix, and minimize ion suppression. However, this approach also introduces variability in metabolite extraction efficiency, impacting reproducibility and sensitivity. To address this, we tested whether pretreatment with different concentrations of FA, prior to MeCN precipitation, could improve metabolite extraction performance. Initially, blood plasma samples (*n* = 5 replicates) were processed with MeCN alone or with FA pretreatment across a range of concentrations (0.1–5%) and analyzed by RPLC-MS in pos. and neg. ionization modes. At 1% FA and above we observed a step-change in the reproducibility of metabolite extraction, an increase in the number of compound-features with %CV < 30 and higher average metabolite intensities (Figure 1a-d). The experiment was repeated multiple times using two separate LC-MS systems (UPLC coupled to Waters Xevo G2-XS QTOFs). Results were consistent across experiment replicates and platforms. Improvements in extraction reproducibility were also observed using serum samples (Figure S1).

**Figure 1:**
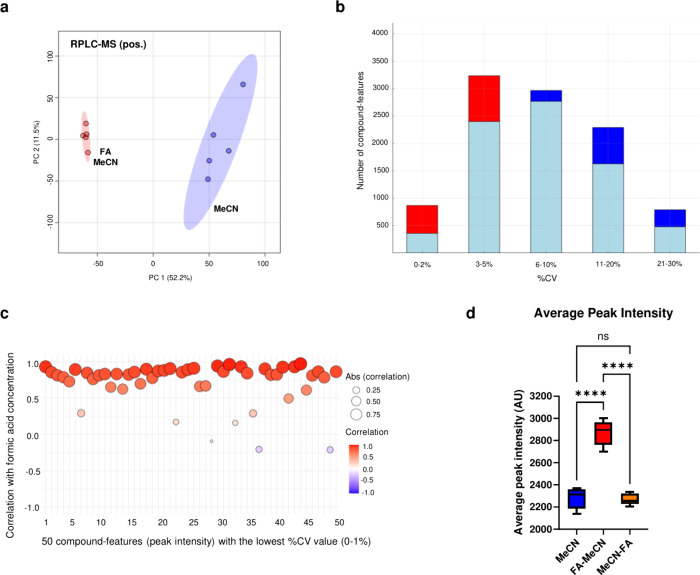
**Impact of formic acid (FA) on plasma extraction efficiency and reproducibility.** (a) PCA plot showing tighter clustering of FA-treated extracts (shaded area = 95% confidence interval). (b) Histogram illustrating the distribution of %CV values for compound-features detected following extraction with 1% FA prior to MeCN (red) or with MeCN only (dark blue). Overlapping features in each ‘%CV bin’ are shown in light blue. (c) Correlation plot showing the relationship between FA concentration and normalized peak intensities for compound-features with low variability (%CV 0–1%, top 50 compound-features). Dot color reflects the direction and strength of the Pearson correlation (blue = negative, red = positive), while dot size corresponds to the absolute correlation value, indicating the strength of association regardless of direction. (d) Box plots comparing average peak intensities (%CV < 30) across three extraction strategies: MeCN alone, FA pretreatment followed by MeCN extraction (FA-MeCN), and postspiking with FA after MeCN extraction (MeCN-FA), (****p < 0.0001; ns = not significant; one-way ANOVA with *post hoc* test). All panels are based on *n* = 5 sample preparation replicates per condition. All analyses were conducted using RPLC-MS in pos. ionization mode.

The markedly tighter clustering of FA+MeCN extracts in pos. ion mode indicated a reduction in technical variance (Figure 1a). Interestingly, FA pretreatment led to an increase in the number of compound-features with %CV < 30% and substantially elevated the proportion with %CV < 5% (Figure 1b), despite a modest reduction in total compound-feature count (*p* = 0.03). Among the 50 most stable compound-features (%CV 0–1%), a strong positive correlation with FA concentration was observed, supporting a concentration-dependent effect (Figure 1c). Pretreatment with FA also resulted in a significant increase in the average ion intensity (*p* < 0.0001; Figure 1d). This trend was consistent across both pos. and neg. ionization modes (Figure S2). Notably, no further gains in intensity or reproducibility were observed with increasing FA concentration above 1% (Figure S3), and total protein removal remained unaffected (*p* = 0.2), indicating that FA pretreatment improved analytical performance without compromising protein precipitation efficiency.

### Lowered pH Fails to Explain FA-Induced Improvements

We hypothesized that increased proton availability in FA-treated samples could be enhancing ionization efficiency, for example promoting [M+H]^+^ formation resulting in higher, and more reproducible, ion abundances. To test this, we compared three extraction approaches using human plasma: (1) MeCN alone (control), (2) 1% FA pretreatment followed by MeCN, and (3) MeCN extraction followed by postspiking with 1% FA. Samples were analyzed by RPLC-MS in both pos. and neg. ionization modes, and using anion-exchange chromatography-MS (AEC-MS). The AEC-MS method incorporated an electrolytic ion suppressor that removed mobile phase cations—including protons—providing a control for FA-induced ionization effects (in neg. ionization mode only; Supporting Information, eMethod 1.5).^20,25^ Our results showed that post-precipitation addition of FA led to improved clustering of metabolite extraction replicates compared to MeCN-only extracts; however, the most consistent clustering was observed when FA was added prior to MeCN precipitation (Figure 2a). Notably, while both FA-based methods reached the same final pH, only pretreatment with FA (FA–MeCN) resulted in significantly higher average ion intensity in RPLC-MS pos. ionization mode (*p* < 0.0001; Figure 1d), whereas no significant difference was detected between MeCN-only and post-FA-spiked samples (*p* = 0.98; Figure 1d). We also tested the addition of FA directly to the extraction solvent to achieve a final concentration of 1% FA in the plasma samples and observed a similar but slightly less reproducible improvement in clustering and signal stability (Figure S4). This approach, however, necessitates a proportionally higher FA concentration in the resulting LC-MS sample to achieve a final concentration of 1% FA relative to the plasma volume. Given these considerations, we favoured FA pretreatment. These findings suggested that the improvements in reproducibility and signal intensity were not solely driven by a reduction in pH introduced by FA or enhanced proton availability leading to enhanced ionization efficiency. Importantly, no detrimental impact on ion intensity was observed in the neg. ionization mode following 1% FA pretreatment (Figure S2), indicating compatibility with both ionization conditions.

**Figure 2:**
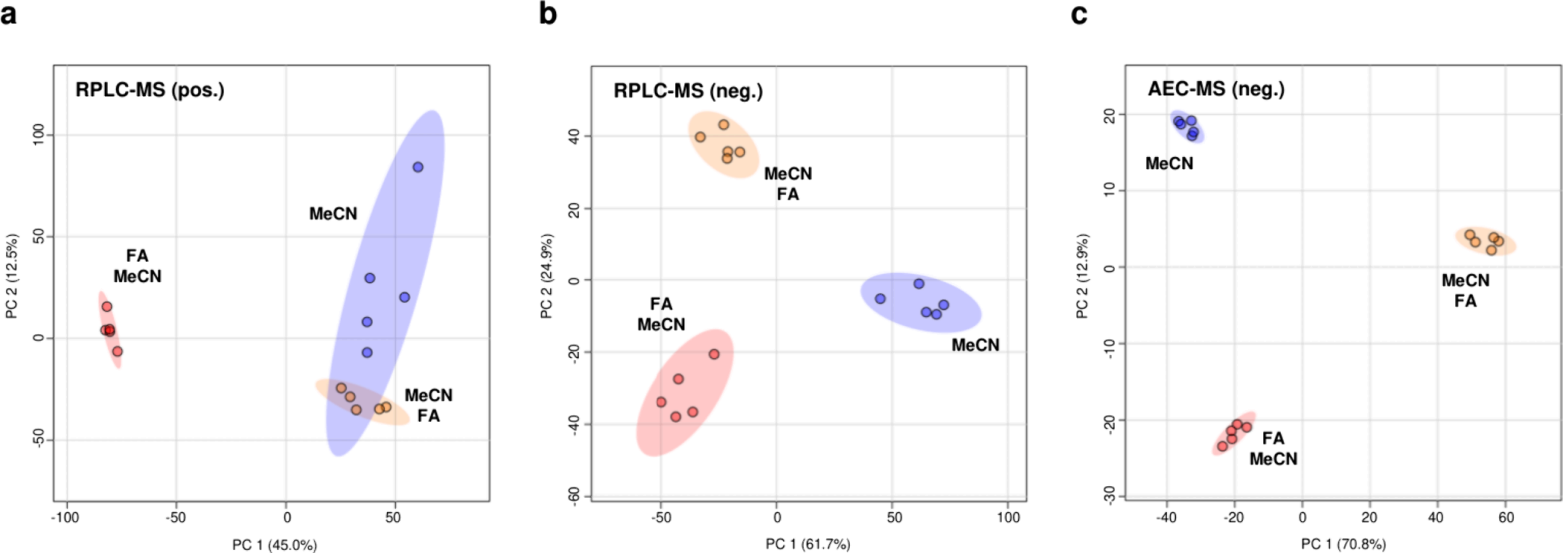
**Reproducibility of extraction workflows across LC-MS platforms.** PCA plots of plasma metabolite extracts prepared using three workflows: MeCN-only (blue), FA pretreatment followed by MeCN (FA-MeCN; red), and MeCN extraction followed by FA addition (MeCN-FA; orange). (a) RPLC-MS (pos. ionization mode), (b) RPLC-MS (neg. ionization mode), and (c) AEC-MS (neg. ionization mode). Each panel shows *n* = 5 replicate extractions from the same plasma aliquot.

In RPLC-MS neg. mode, FA pretreatment (FA+MeCN) exerted minimal effect on sample clustering (Figure 2b); however, the average ion intensity profile mirrored that of pos. ionization mode, with FA added prior to MeCN yielding significantly higher ion intensities than MeCN alone or MeCN+FA (Figure S2). This pattern was also observed in AEC-MS (neg. ionization mode), where all extraction groups showed tighter replicate clustering, likely reflecting improved retention and separation of ionic and polar metabolites (Figure 2c, Figure S5). Although FA addition during sample preparation substantially lowered pH (∼2.5) in the sample vial, it is unlikely the pH would be substantially different from the mobile phase at the point of ionization. H^+^ would be rapidly and substantially diluted by mobile phase flow through the system. Notably, in neg. mode, all three extraction methods (MeCN, FA-MeCN, and post-FA) formed distinct PCA clusters, despite comparable average ion intensities between MeCN-only and post-FA extracts. These findings suggest that FA addition prior to precipitation alters the physicochemical properties of the extract, thereby affecting ionization characteristics and downstream multivariate clustering.

### Addition of FA Minimally Impacted Adduct Formation

To assess whether addition of FA altered adduct formation, we examined FA and MeCN adducts in both pos. and neg. ionization modes across both chromatographic methods (Tables S1–S3). Adducts were automatically annotated using peak picking and deconvolution with Progenesis QI software and also manually verified. No notable differences in adduct profiles (the proportion of different adduct forms) were observed between FA-treated and untreated samples. As the mobile phase already contained 0.1% FA, formate-related adducts were present in all experimental groups, but the additional FA introduced during extraction, did not significantly alter the adduct profile (*p* > 0.05). It was also observed that addition of FA (before or after MeCN precipitation) had no detectable effect on sodium or potassium adduct formation, indicating that alkali metal ion clustering remained unaffected. Taken together, these results confirmed that FA treatment did not systematically alter adduct behavior under the conditions used.

### Disrupting Protein–Metabolite Interactions

Our experiments consistently revealed enhanced sample clustering and increased total ion abundance in plasma samples treated with FA prior to MeCN extraction. At physiological pH, plasma proteins have been shown to maintain native structures and readily form noncovalent complexes with small molecules.^8^ Furthermore, endogenous proteins such as serum albumin and lipoproteins are also known to play a physiological role as metabolite carriers, aiding the circulation of hydrophobic and hydrophilic compounds, including hormones, fatty acids, lipids, and xenobiotics.^8,21^ We hypothesized that during protein precipitation, noncovalent interactions may lead to the sequestering of a proportion of protein-bound metabolites, impacting their extraction efficiency, and that FA pretreatment may disrupt these interactions prior to sequestration via precipitation, leading to improved metabolite release and extraction efficiency.

To test this hypothesis, plasma samples were incubated with additional bovine serum albumin (BSA, 150 μM) and extracted with or without prior FA treatment (Supporting Information, eMethod 1.1). In RPLC-MS pos. ionization mode, BSA addition led to a marked increase in the number of compound-features detected in FA-treated samples compared to those extracted with MeCN alone (Figure 3a). This supported the hypothesis that metabolites may be bound to protein in serum and plasma and that prior FA treatment would help to disrupt protein–metabolite interactions. We observed similar results for RPLC-MS neg. mode (Figure S6a); however, no increase in compound-features was seen with AEC-MS (Figure S6b). As AEC-MS selectively detects small, highly polar and ionic compounds, this suggested susceptable metabolites may be more hydrophobic and/or possibly of higher molecular weight. This is consistent with the physicochemical characteristics of compounds previously reported bound to proteins *in vivo*, including lipids and fatty acids. This interpretation was further supported by Student’s *t* test results from the plasma extraction comparison, which showed that the most significantly altered compound-features were predominantly increased in FA-treated samples (Table S4). Their *m*/*z* values and retention times (Table S4) suggested they were primarily hydrophobic and ‘lipid-like’, consistent with the release of more hydrophobic compounds from protein binding sites. To assess whether the observed effects were influenced by differences in protein removal, potentially impacting ion suppression and signal-to-noise ratios, we compared residual protein content between MeCN-only and FA pretreated samples; no significant differences were observed (150 ± 30 μg/mL vs 170 ± 20 μg/mL; *p* = 0.2).

**Figure 3:**
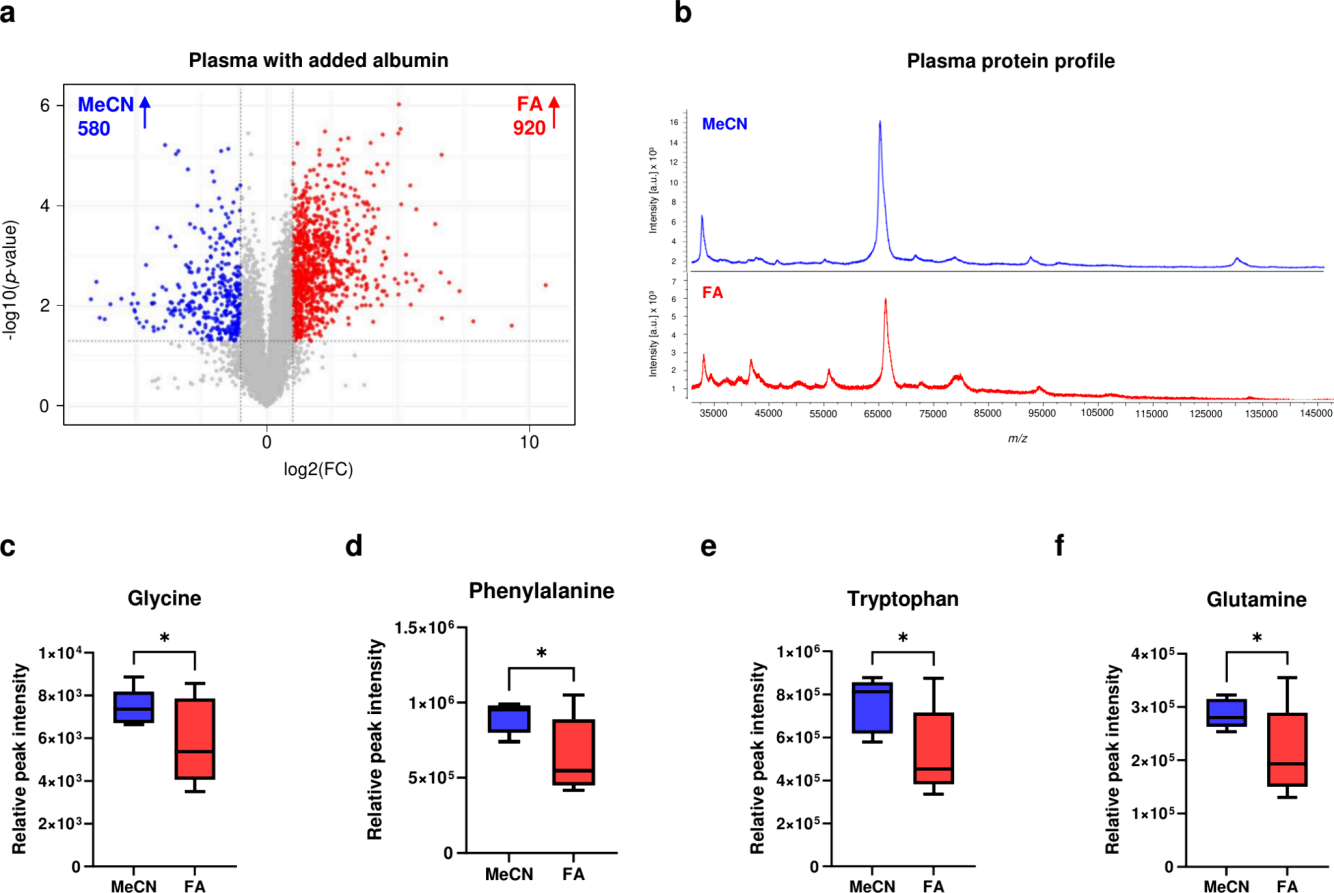
**Effects of formic acid–protein interactions on metabolite extraction efficiency.** (a) Volcano plot showing altered compound-features between acidified and unacidified plasma with additional albumin (*c* = 7.0 g/dL) extractions (*t* test, *p* < 0.05, uncorrected; RPLC-MS (pos. ionization mode)). (b) MALDI-MS spectra of the resuspended plasma protein pellet following extraction with acetonitrile (MeCN, blue) and formic acid–acetonitrile (FA-MeCN, red). Both spectra are dominated by albumin peaks, with no significant differences observed. (c–f) Amino acids significantly altered between MeCN and FA-MeCN plasma extracts (HILIC-MS data): (c) glycine (*p* = 0.02), (d) phenyl­alanine (*p* = 0.01), (e) tryptophan (*p* = 0.01), and (f) glutamine (*p* = 0.02). All extractions correspond to *n* = 5 samples from the same plasma aliquot.

Finally, to evaluate whether FA treatment prior to protein precipitation led to protein degradation we analyzed the protein content of the post-extraction pellets. Plasma and aqueous albumin solutions were extracted using MeCN or FA+MeCN and the resulting pellets were reconstituted and analyzed by MALDI-MS. In both cases, intact serum albumin was observed and while no major differences in protein profiles were detected, the MALDI spectra suggested that FA-treatment may have slightly enhanced albumin denaturation, as indicated by minor shifts in high (30–200 kDa; Figure 3b, Figure S7) and low (4–20 kDa; Figures S8, S9) molecular weight ranges. MALDI analysis of the corresponding supernatants confirmed the absence of detectable peaks corresponding to peptide or protein for both extraction conditions, indicating that degradation products were not observable in the supernatant.

To confirm that FA pretreatment did not lead to the release of proteinogenic amino acids via protein hydrolysis, we performed HILIC-MS analysis on MeCN-only and FA-MeCN extracts to compare free amino acid levels. Of the 20 main proteinogenic amino acids, 15 were detected (aspartic acid, glutamic acid, threonine, cysteine, and asparagine were not observed; Table S5). For most amino acids, FA pretreatment had no significant effect on their relative abundances. Interestingly, levels of glycine (*p* = 0.02; Figure 3c), phenyl­alanine (*p* = 0.01; Figure 3d), tryptophan (*p* = 0.01; Figure 3e), and glutamine (*p* = 0.02; Figure 3f) were significantly lower in abundance in FA-treated samples, with tyrosine showing borderline significance (*p* = 0.09). These findings provided no indication that FA pretreatment promoted the release of free amino acids via protein hydrolysis or related mechanisms.

### Case Study 1: OPTIMA

To evaluate the translational potential of FA-enhanced extraction into clinical sample analysis, we applied both MeCN-only and FA-MeCN protocols to serum samples from the OPTIMA study, a dementia case-controlled study.^14,22^ While method development was performed in plasma, comparable improvements in reproducibility and PCA clustering with FA pretreatment, were also observed in both standard serum and deuterated phosphate-buffered serum matrices (Figure S1).^23^ The OPTIMA sample analysis focused on a subset of 63 patients with mild to moderate cognitive impairment, comprising 34 individuals who later developed vascular dementia (VaD) and 29 who progressed to Alzheimer’s disease (AD). Samples were analyzed using RPLC-MS in pos. ionization mode, and group separation was assessed using OPLS-DA models comparing AD and VaD outcomes. As expected for early stage differentiation, the overall model performance was modest. Using MeCN-only extraction, the classification accuracy reached 53.5 ± 7.3% (Figure 4a), which was nonetheless significantly above the accuracy of a random model ensemble (*p* < 0.001). Notably, the pretreatment with FA prior to MeCN extraction improved classification accuracy to 58.6 ± 6.0% (Figure 4b). These results were based on 1000 cross-validated models per condition, with the improvement in classification accuracy reaching statistical significance (*p* = 4.31 × 10^–6^, Figure 4c). Similar improvements were observed in sensitivity (*p* = 4.7 × 10^–4^) and specificity (*p* = 9.25 × 10^–6^, Figure S10). These findings align with our earlier observations that FA pretreatment enhances reproducibility, increases metabolite signal intensity, and improves the detection of low-variance features across the metabolome.

**Figure 4:**
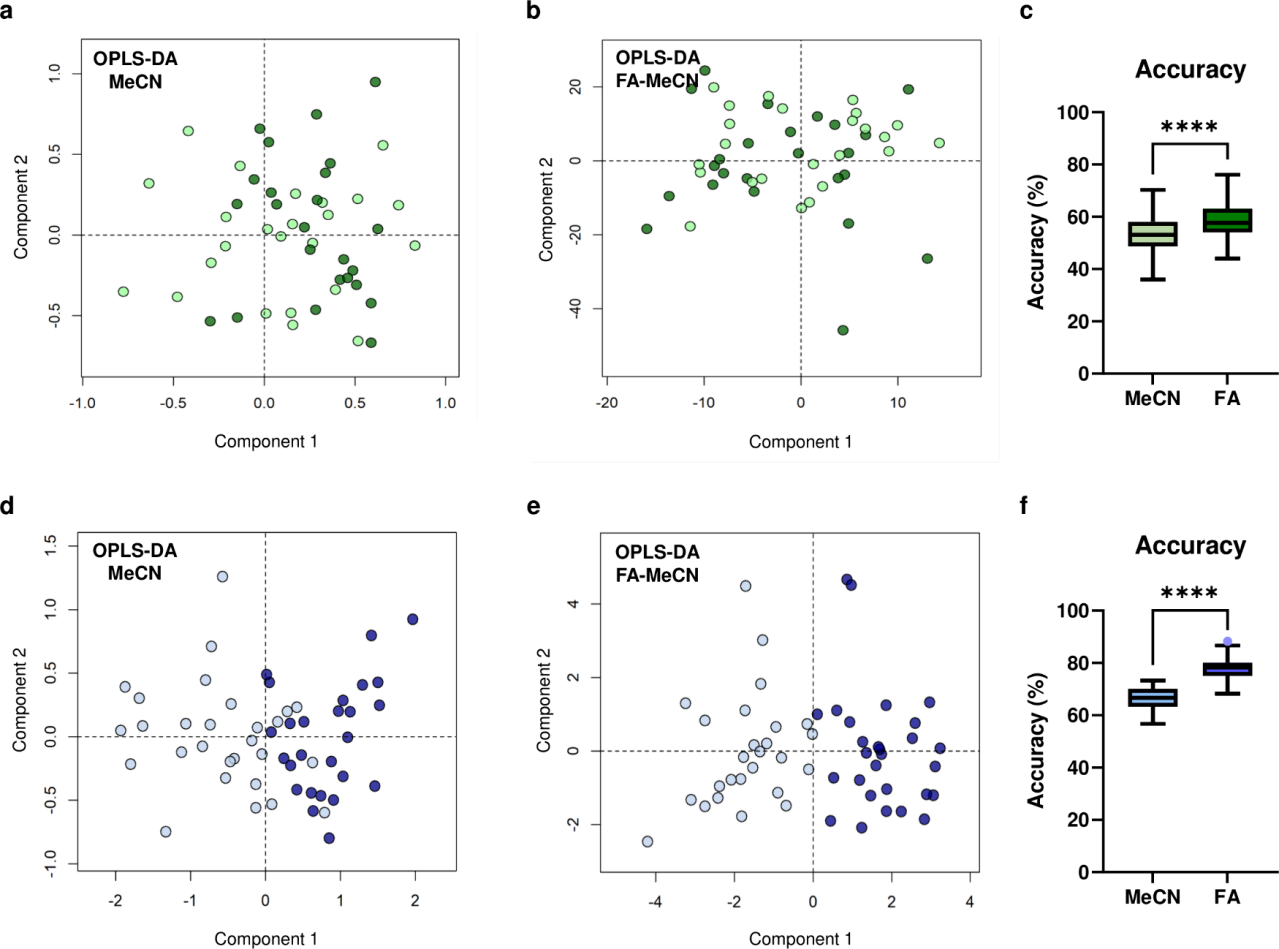
**Comparison of extraction methods across two independent clinical cohorts.** Serum samples from the OPTIMA (a–c) and VITACOG (d–f) studies were extracted using either MeCN (a, d) or FA-enhanced (b, e) workflows. (a, b) OPLS-DA score plots for OPTIMA (light green: Alzheimer’s disease; dark green: vascular dementia). (d, e) OPLS-DA score plots for VITACOG (light blue: B vitamin treatment; dark blue: placebo). (c, f) Classification accuracy from 1000 cross-validated models. In both cohorts, FA-based extraction consistently improved classification performance (****p < 0.0001, Kolmogorov–Smirnov test).

### Case Study 2: VITACOG

To demonstrate the consistency of improvements observed in multivariate models, the two extraction methods were also evaluated in a more clearly defined cohort from a different clinical study focused on mild cognitive impairment: the VITACOG study, where more pronounced metabolic differences were anticipated.^15,24^ The analysis used serum samples prepared in deuterated phosphate buffer from 30 individuals with mild cognitive impairment who had received B vitamin supplementation (previously identified as treatment responders), alongside 30 placebo-treated controls. Consistent with the OPTIMA findings, FA-enhanced extraction outperformed MeCN-only (Figure 4d,e), yielding significantly higher classification accuracy (77.9 ± 3.7% vs 66.4 ± 4.0%, *p* = 1.45 × 10^–52^, Figure 4f), sensitivity (80.2 ± 5.0% vs 70.3 ± 6.1%, *p* = 4.18 × 10^–52^), and specificity (78.2 ± 5.6% vs 67.2 ± 6.3%, *p* = 2.13 × 10^–27^, Figure S10).

In both cohorts, VIP-ranked features showed minimal overlap between extraction methods, indicating that each captures distinct regions of the serum metabolome (Tables S6–S9). The FA-based protocol, which consistently achieved higher classification accuracy, yielded a smaller but more consistent selection of discriminative features. In contrast, MeCN-only extraction identified a broader array of features with lower individual contributions to group separation, suggesting a more diffuse, “noisy” signal. Notably, some features were uniquely detectable with FA+MeCN extraction, indicating method-specific enrichment (Tables S10–S12) likely driven by metabolite class–dependent responses influenced by polarity, stability, and matrix interactions. The FA+MeCN approach also yielded significantly larger FC between groups (*p* = 0.007; Tables S10, S12), reflecting improved biological resolution and enhanced detection of treatment-related metabolites. These findings align with our pooled plasma and serum experiments, where MeCN-only extraction yielded a slightly higher total number of compound-features (*p* = 0.03) but with lower reproducibility and signal intensity. In contrast, FA pretreatment enabled more consistent recovery of analytically robust features, including compounds undetectable with MeCN alone. The improved performance may also result from greater matrix stabilization, as acidification standardizes sample pH, reducing heterogeneous chemical interconversions, degradation, and adduct formation. Collectively, the results from both clinical studies illustrated a consistent benefit from sample pretreatment with FA.

## Conclusions and Limitations

This observational study demonstrated that pretreatment of plasma and serum with 1% FA prior to MeCN extraction reproducibly enhanced analytical performance using untargeted metabolomics, particularly using RPLC-MS in pos. ionization mode. FA pretreatment consistently led to increased compound abundances, improved sample clustering in both supervised and unsupervised models, and reduced %CV values across detected compound-features. These improvements translated into greater classification accuracy in two independent clinical studies, supporting the translational potential of this simple, low-cost optimization of the method.

While the specific mechanisms underlying the observed effects remain to be fully elucidated, experiments using multiple analytical platforms and sample preparation controls, suggest that several physiochemical processes may contribute to the metabolome-wide changes observed. These include matrix pH standardization, stabilization of labile metabolites, and disruption of protein–metabolite interactions. It is important to note, however, that FA may differentially affect individual analytes by influencing their stability, ionization efficiency, or physicochemical properties. Such effects could be analyte- and/or platform-dependent, and targeted validation is recommended if applying this sample preparation approach to alternative matrices or compound-classes. For example, in lipidomics workflows, the impact of acidification on lipid recovery or chemical stability may vary depending on the specific lipid sub-classes involved and should be carefully evaluated.

Taken together, our findings demonstrate the utility of FA pretreatment as an effective and scalable approach to improve reproducibility, sensitivity, and discriminatory power using untargeted metabolomics workflows, particularly in the context of clinical research, personalized medicine, and biomarker discovery, where metabolic changes can be subtle and challenging to detect and where improved sensitivity and reproducibility can have a significant impact on clinical outcomes.

## Supplementary Material



## Data Availability

The test data set generated during method development is publicly available through the Oxford Research Archive (ORA) at DOI: 10.5287/ora-nrormvy24. Anonymized data from the clinical studies will be made available upon request from any qualified investigator.

## Notes

Detailed experimental protocols for plasma and serum extraction, mass spectrometry workflows (RPLC-MS, AEC-MS, HILIC-MS, MALDI-TOF MS), data processing, and statistical analyses; descriptions of clinical cohorts (OPTIMA and VITACOG); and additional figures and tables presenting PCA analyses, box plots, MALDI spectra, adduct summaries, and VIP features from multivariate models (PDF)

## Notes

**Ethics Approval and Consent to Participate.** The trial, registered as VITACOG under the title “Homocysteine and B Vitamins in Cognitive Impairment” (ISRCTN 94410159), was conducted in accordance with the principles outlined in the Declaration of Helsinki. Ethical approval was granted by the local NHS research ethics committee (COREC 04/Q1604/100). For OPTIMA, protocols were approved by the Central Oxford Ethics Committee, number 1656. All participants provided written consent after information regarding the study protocol was provided to the participants and, in cases of moderate to severe dementia, also to a caregiver.
