# Supplementary material for: Formic Acid Pretreatment Enhances Untargeted Serum and Plasma Metabolomics
Source: Anal Chem. 2025 Oct 7;97(42):23014–21. doi: 10.1021/acs.analchem.5c03725 (PMC12573228; doi:10.1021/acs.analchem.5c03725)
Supplement: Supplementary file 1 [file ac5c03725_si_001.pdf]

Supplementary Information

Formic Acid Pre-treatment Enhances Untargeted Serum and Plasma Metabolomics

Authors: Tereza Kacerova[1], Elisabete Pires[1], Abigail Dixon[1], Rachel Williams[1], Isabelle Legge[1], Mia Hippisley[1], Abi G. Yates[1,2], A. David Smith[2], Daniel C. Anthony[2], Fay Probert[1], James S. O. McCullagh[1]*

[1] Chemistry Research Laboratory, Department of Chemistry, University of Oxford, Oxford OX1 3TA, UK

[2] Department of Pharmacology, University of Oxford, Oxford OX1 3QT, UK

Corresponding author: James S. O. McCullagh james.mccullagh@chem.ox.ac.uk

Table of contents

1 eMethods

1. 1 Plasma metabolite extraction **S4**

1. 2 Clinical serum samples (VITACOG and OPTIMA) metabolite extraction **S4**

1. 3 Total protein content measurement **S4**

1.4 Reversed-Phase Ultra Performance Liquid Chromatography (RPLC)-MS Analysis **S5**

1.5 Anion-Exchange Chromatography (AEC)-MS Analysis **S5**

1.6 Hydrophilic Interaction Liquid Chromatography (HILIC)-MS Analysis **S5**

1.7 MALDI-TOF MS Analysis **S6**

1.8 Data Processing **S6**

1.9 Statistical analysis **S6**

2 eMethods – Clinical Cohorts

2.1 OPTIMA **S8**

2.2 VITACOG **S8**

2.3 Participants consent and trials registration **S8**

3 SI Results – SI Figures and SI Tables

Figure S1. PCA analysis of FA–MeCN versus MeCN-only extraction in serum. **S9**

Figure S2. Box plots comparing average peak intensities across three extraction strategies. **S10**

Figure S3. PCA plot of RPLC–MS (pos. ionisation mode) data comparing protein precipitation using MeCN alone versus FA pre-treatment at increasing concentrations (1%, 2%, and 5% v/v). **S10**

Figure S4. PCA plot of plasma metabolite extracts prepared using three different acidification strategies for RPLC-MS in positive ionisation mode. **S11**

Figure S5. Impact of formic acid on plasma extraction efficiency and reproducibility. **S12**

Table S1. Summary of common adducts detected in RPLC-MS (positive ion mode) **S13**

Table S2. Summary of common adducts detected in RPLC-MS (negative ion mode) **S13**

Table S3. Summary of common adducts detected in AEC-MS (negative ion mode) **S14**

Figure S6. Effects of Formic Acid–Protein Interactions on Metabolite Extraction Efficiency. **S15**

Figure S7. MALDI-MS spectra of the albumin protein pellet (high mass range) **S16**

Figure S8. MALDI-MS spectra of the plasma protein pellet (low mass range) **S17**

Figure S9. MALDI-MS spectra of the albumin protein pellet (low mass range) **S18**

Table S4. Compound-features with the lowest *p*-values in unpaired Student's t-tests comparing plasma extracts prepared with FA+MeCN versus MeCN alone **S19**

Table S5. Comparison of proteinogenic amino acid levels (HILIC-MS). **S20**

Figure S10. Formic acid–based extractions enhance the discriminatory capacity of supervised multivariate statistical models. **S21**

Table S6. List of the variables importance in projection identified from the AD vs VaD model (OPTIMA) for the MeCN-based extractions. **S22**
Table S7. List of the variables importance in projection identified from the AD vs VaD model (OPTIMA cohort) for the FA-MeCN-based extractions. **S23**
Table S8. List of the variables importance in projection identified from the B vitamin-treated vs placebo model (VITACOG cohort) for the MeCN-based extractions. **S24**
Table S9. List of the variables importance in projection identified from the B vitamin-treated vs placebo model (VITACOG cohort) for the FA-MeCN-based extractions. **S25**
Table S10. Comparison of compound-features extracted using FA-based and pure acetonitrile-based protocols. **S26**
Table S11. Compound-features with the highest VIP scores in the comparison between the vitamin B-treated (VB) and placebo (P) groups, based on FA-extracted data. **S27**
Table S12. Compound-features with the highest VIP scores in the comparison between the vitamin B-treated (VB) and placebo (P) groups, based on FA-extracted data. **S28**

1 eMethods

1. 1 Plasma metabolite extraction

For all experiments, fresh human plasma from Research Donors (Cambridge Bioscience, UK) was used. The plasma aliquots were thawed at 4°C. All samples were prepared in replicates of five and each MS injection was repeated twice in random order. Metabolites from human plasma were extracted by adding formic acid (Merck, ≥95%) to achieve the required concentration (0.01-5.00% were tested). The samples were then vortexed, extracted in acetonitrile (1 : 2.33 (v/v)), and centrifuged (16 000 x g; 15 min, 4°C). The supernatant was transferred into the total-recovery MS vials. For albumin experiments, bovine serum albumin (Merck, ≥98%) was either dissolved in Milli Q water or added to plasma (incubation time 3h) to achieve a final concentration of 150 µM in plasma.

1. 2 Clinical serum samples (VITACOG and OPTIMA) metabolite extraction

The serum aliquots were thawed at 4°C and the samples were visually checked for any protein precipitation. All serum samples were processed for a parallel NMR and multi-LC-MS analysis, according to well-established protocol. 405 µL of the sample was then diluted with 50 µL of 75 mM sodium phosphate buffer prepared in D_2O (pH 7.4). For the first sample set (acetonitrile) 75 µL of the resulting sample was mixed with 200 µL of acetonitrile to precipitate any residual protein. The samples were vortexed for 1 min, centrifuged (16 000 x g; 15 min, 4°C), and the supernatant was transferred into total-recovery MS vials. For the second sample set; 0.78 µL of formic acid was added to the mixture of serum and deuterated phosphate buffer. The samples were vortexed for 1 min and the resulting sample was mixed with 200 µL of acetonitrile to precipitate any residual protein. The samples were vortexed for 1 min, centrifuged (16 000 x g; 15 min, 4°C), and the supernatant was transferred into total-recovery MS vials.

1. 3 Total protein content measurement

Total protein concentrations for each extraction method were quantified using the Pierce™ Rapid Gold BCA Protein Assay Kit-1 (Thermo Fisher Scientific, UK). Supernatants (µg μL^{-1}) were mixed with working reagent in a 1:20 ratio (sample:reagent), and absorbance at 480 nm was measured using a ClarioStar Plus microplate reader (BMG LABTECH, Germany) with an LVis plate.

1.4 Reversed-Phase Ultra Performance Liquid Chromatography (RPLC)-MS Analysis

C18 reversed-phase analysis was performed using an Acquity UPLC liquid chromatograph (produced by Waters, UK) system with a gradient elution program coupled directly to a high-resolution tandem mass spectrometer, Xevo G2-XS QTOF. A 5 µL partial loop injection was used for all analyses with pre- and post-injection wash programs. The technical details have been previously published in [Kacerova T., *et al., Anal Chim Acta*. 2025; 1356: 343979. doi: 10.1016/j.aca.2025.343979].

1.5 Anion-Exchange Chromatography (AEC)-MS Analysis

Anion-exchange chromatography-MS (AEC-MS) analysis was carried out using a Dionex ICS-5000+ high-pressure ion chromatography system with a continuously regenerated trap column, Dionex ERS 500e suppressor and AS11-HC (2 × 250 mm, 4 µm) column, all from Dionex (Sunnyvale, CA, USA). The technical details have been previously published in [Walsby-Tickle J.., *et al., Commun Biol*. 2020; 3: 247. doi: 10.1038/s42003-020-0957-6].

1.6 Hydrophilic Interaction Liquid Chromatography (HILIC)-MS Analysis

The samples were analysed using an Atlantis Premier BEH Z-HILIC column (1.7µm, 2.1 x 150 mm) on an Ultimate 3000 LC system coupled to an Orbitrap Exploris™ 240 mass spectrometer in H-ESI mode. Eluent A was 100% Acetonitrile and Eluent B was 5mM Ammonium Formate in Type-1 water. A gradient program was used as follows: 0 mins – 5% B; 2 mins – 5% B; 6 mins – 15% B; 14 mins – 60% B; 15 mins – 60% B; 16 mins – 5% B; 30 mins – 5% B at a flow rate of 0.4 mL/min. The column temperature was 50 °C and the autosampler was kept at 4 °C with an injection volume of 5 µL.

The samples were analysed in positive ion mode with a top 10 data dependent acquisition MS2 approach. The spray voltage was 3500 V and the gas flow rates were 25, 8, and 0 for the sheath gas, aux gas, and sweep gas respectively. The ion transfer tube and vapouriser temperatures were both 300 °C. The full scan resolution was 60,000 with a scan range of 60-900 *m/z*. The microscans were set to two with the AGC target set to 5e6 and the maximum injection time set to 120 ms. The ddMS2 resolution was 15,000 with an isolation window of 2 *m/z* and the normalised HCD collision energy was 35%. 2 microscans were used with the AGC target set to 1e5 and the maximum injection time set to 80 ms.

1.7 MALDI-TOF MS Analysis

Sinapinic acid (SA) was prepared at a concentration of 10 mg/mL in a solvent mixture comprising 50% (v/v) acetonitrile, 50% (v/v) water, and 0.3% (v/v) trifluoroacetic acid (TFA). The solution was vortexed for 1 minute, sonicated for 15 minutes, and vortexed again for 1 minute to ensure complete dissolution.

For MALDI-TOF MS analysis, 3 µL of each sample was mixed with 3 µL of the SA matrix solution. A volume of 1.5 µL of the resulting mixture was spotted onto a 384-position ground steel MALDI target plate and air-dried at room temperature. Calibration spots containing bovine serum albumin (BSA, 66,414 Da) were prepared using the same protocol. Mass spectra were acquired using a MALDI Autoflex Speed mass spectrometer (Bruker Daltonics, Bremen, Germany), equipped with an Nd:YAG laser (355 nm) operating at a repetition rate of up to 2 kHz. The instrument was operated in linear positive ion mode under the following settings: mass range 30–210 kDa, sampling rate 0.16 GS/s, 1000 laser shots per spectrum, 100% laser power, 100 Hz laser frequency, and 25 kV detector gain. External mass calibration was performed using BSA. Spectral data were processed using FlexAnalysis software (Bruker Daltonics).

1.8 Data Processing

The untargeted metabolomics data were processed in Progenesis QI (Nonlinear Dynamics, Waters, Elstree, UK), and the detected compound-features were compared between the different extraction methods, primarily focusing on the total number of detected compound-features and their associated % coefficient of variance (%CV) for evaluating the efficacy of sample preparation efficiency across independent datasets.

Metabolite identification was based on matching multiple experimental data to metabolite databases. In-house libraries of over 450 (IC-MS) and 150 (RPLC-MS) authentic metabolite standards with matching of multiple independent measurements under the same experimental conditions to provide Level 1 identifications (e.g. retention time (Rt error < 2 min; accurate-mass < 5 ppm (IC-MS) and 20 ppm (RPLC-MS); isotope and fragmentation patterns > 90%).

1.9 Statistical analysis

The %CV filtered (30% cut-off) data exported from Progenesis were processed in MetaboAnalyst 6.0 and R software 4.2.1 (R Foundation for Statistical Computing, Vienna, Austria), using the *corrr*, *ggplot2*, *ggpubr*, and *dplyr* packages. The datasets were sum normalised, standard scaled and log transformed. Univariate

statistical analysis included determining fold change (FC) and *t* test *p* values between different extraction methods for compound-features and combined in volcano plots. The FC threshold was set to 2 and the *p* value cut-off was set to 0.05 to determine significance. The data were projected using an unsupervised principal component analysis (PCA), which allowed the similarities within the individual datasets to be shown. The significance cut-off was set at $p < 0.05$ for all data sets. To maximise the likelihood of identifying potential differences between extraction methods, multiple testing corrections such as false discovery rate (FDR) were generally not applied.

To assess metabolomic differences between treated individuals and placebo controls, we applied logistic regression with elastic net regularisation and orthogonal partial least squares discriminant analysis (OPLS-DA), adapted from previously published work. Each data point in the resulting multivariate plots represents the serum metabolomic profile of a specific individual with mild cognitive impairment (MCI). The dataset was split into two classes, and feature selection was performed on the training set during each fold. Elastic net regularisation ($\alpha = 0.5$) with cross-validation was used to determine the optimal regularisation parameter (λ), and features were selected based on non-zero coefficients from the regularised logistic regression model.

For OPLS-DA, 10-fold cross-validation with repetition and permutation testing was used to mitigate overfitting. Each model was tested using an independent subset of samples excluded from the training set, allowing for the assessment of model accuracy, sensitivity, and specificity. To validate feature selection and classification performance, randomised null models were generated by shuffling class labels. If the final model ensemble significantly outperformed 1000 null models (two-sided Kolmogorov–Smirnov test, $p < 0.05$), discriminatory compound-features were extracted by averaging Variable Importance in Projection (VIP) scores across models. Features were ranked within each model, and their overall importance was calculated as the frequency of selection divided by the average rank, prioritising variables that were consistently selected and highly ranked across iterations.

2 eMethods – The description of clinical cohorts

2.1 OPTIMA

The Oxford Project to Investigate Memory and Ageing (OPTIMA) is a longitudinal study of cognitive decline in ageing individuals. For this analysis, we used a subset of 29 Alzheimer's disease (AD) and 34 vascular dementia (VaD) patients from the OPTIMA cohort, matched for Mini-Mental State Examination (MMSE) scores above 20, indicating mild to moderate cognitive impairment. These were archived serum samples from a subsample previously selected for omics analyses, used here as a proof-of-concept to evaluate and optimise the extraction protocols. For full study report, refer to the following study: [Razay G., *et al., Dement Geriatr Cogn Disord*. 2009; 28: 70. doi: 10.1159/000230877].

2.2 VITACOG

The VITACOG trial was a randomised, placebo-controlled study investigating the effects of B-vitamin supplementation on brain atrophy in individuals with mild cognitive impairment. For this analysis, we selected stored serum samples from 30 participants who received B-vitamin treatment and showed a slowed rate of brain atrophy, and 30 placebo-treated individuals who exhibited accelerated atrophy over the study period. These represent extreme phenotypes within the trial population and were used to explore the performance of the extraction protocols under contrasting biological conditions. For full study report, refer to the following study: [Smith AD., *et al., PLoS One*. 2010; 5: 12244. doi: 10.1371/journal.pone.0012244].

2.3 Participants consent and trials registration

The VITACOG trial was conducted according to the principles described in the Declaration of Helsinki. The study was approved by a local NHS research ethics committee (COREC 04/Q1604/100). Each involved individual provided written consent for their participation. For OPTIMA, full ethics committee approval was obtained for collection of clinical data (Frenchay REC Ref 09/H0107/9) and for use of brain tissue for research (Oxford REC 07/H0606/85 for use of tissue in the Thomas Willis Oxford Brain Collection).

3 Results

FA pre-treatment improves extraction reproducibility and efficiency in serum and phosphate-buffered serum

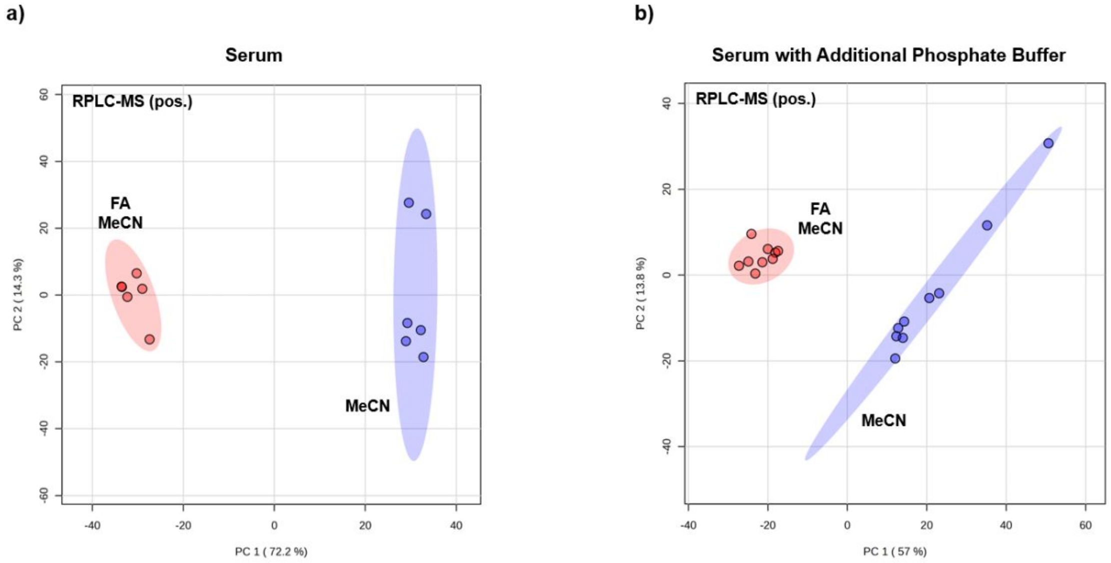

SI Figure 1. PCA analysis of FA–MeCN versus MeCN-only extraction in serum and phosphate-buffered serum. PCA plots showing the effect of extraction method on sample clustering in **(a)** unmodified serum and **(b)** serum supplemented with deuterated phosphate buffer. Samples were extracted using either MeCN-only (blue) or FA pre-treatment followed by MeCN (FA–MeCN; red) and analysed by RPLC-MS in pos. ionisation mode. Each panel represents $n = 5$ replicate extractions from the same serum pool.

Mechanistic insights: extraction effects

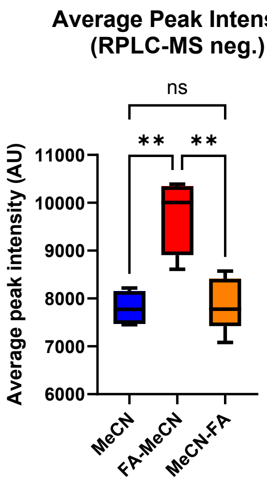

SI Figure 2. Box plots comparing average peak intensities (%CV < 30) across three extraction strategies: MeCN alone, FA pre-treatment followed by MeCN extraction (FA–MeCN), and post-spiking with FA after MeCN extraction (MeCN–FA), (**p < 0.01; ns = not significant; one-way ANOVA with *post hoc* test). The panel is based on n = 5 sample preparation replicates per condition. The analysis was conducted using RPLC-MS in neg. ionisation mode.

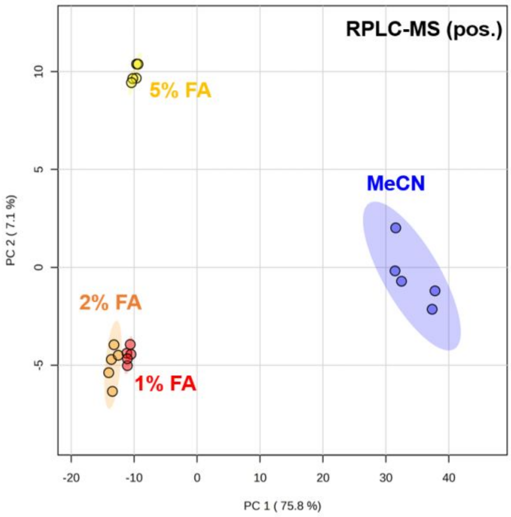

SI Figure 3. PCA plot of RPLC–MS (pos. ionisation mode) data comparing protein precipitation using MeCN alone versus FA pre-treatment at increasing concentrations (1%, 2%, and 5% v/v). While FA pre-treatment markedly improves clustering compared to MeCN alone, higher concentrations (>1%) do not result in further gains in reproducibility. Based on these findings, 1% FA was selected as the optimal concentration (considering also instrument compatibility, as FA is further diluted in the mobile phase prior to entering the ion source).

Mechanistic insights: extraction effects

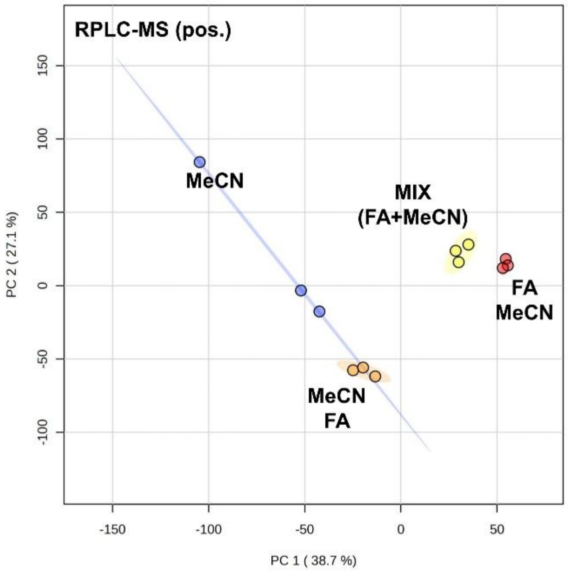

SI Figure 4. PCA plot of plasma metabolite extracts prepared using three different acidification strategies for RPLC-MS in positive ionisation mode. Extraction with MeCN alone (blue), FA pre-treatment followed by MeCN (FA–MeCN; red), and a MeCN solvent containing 1% FA (MIX; yellow) were compared. Both "direct" FA-based methods showed improved clustering relative to MeCN alone, indicating enhanced reproducibility. The MIX approach yielded similar clustering to FA-MeCN.

Mechanistic insights: extraction effects

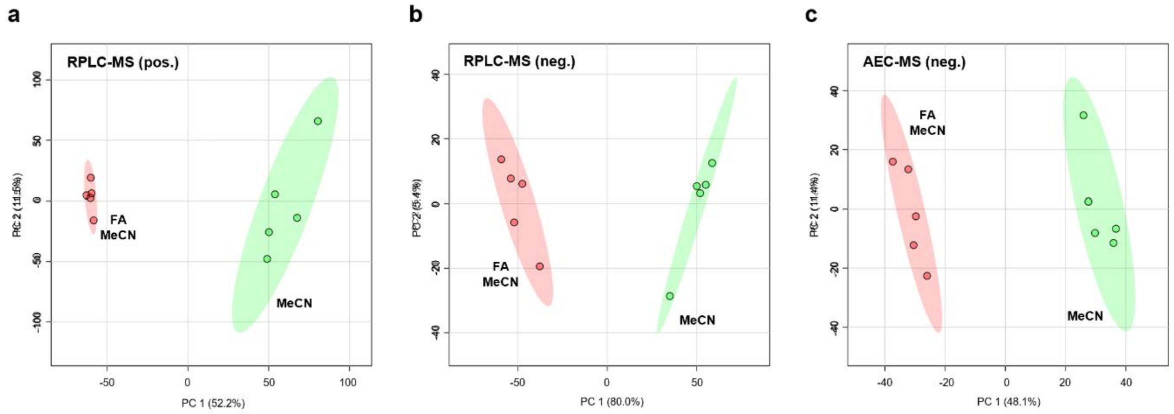

SI Figure 5. Impact of Formic Acid on Plasma Extraction Efficiency and Reproducibility. (a–c) PCA plots highlighting clustering patterns between acetonitrile (MeCN, green) and FA and acetonitrile (FA-MeCN; red) extractions. (a) RPLC-MS (pos. ionisation mode), a significant impact of FA treatment on the extraction reproducibility (i) RPLC-MS (neg. ionisation mode), (j) AEC-MS (neg. ionisation mode). All extractions were prepared in five replicates.

Assessment of adduct formation following formic acid extraction

Adduct profiles were evaluated using the *Progenesis QI* software (Nonlinear Dynamics, Waters, Elstree, UK), which applies automated peak picking followed by adduct deconvolution based on accurate mass differences and isotope pattern matching. The analysis included typical adducts observed in electrospray ionisation, and the assignment was carried out with the default adduct library for both positive and negative ion modes, with tolerance settings of 5 ppm for precursor mass and 20 ppm for fragment ions. Only features consistently detected across replicates (%CV<30) were included in the final comparison.

SI Table 1. Summary of common adducts detected in **RPLC-MS (positive ion mode)** following extraction with either MeCN alone or 1% formic acid (FA) followed by MeCN. The table includes the theoretical mass shifts ($\Delta m/z$) for each adduct relative to the neutral monoisotopic mass (M), along with the number of features assigned to each adduct using Progenesis QI. Adducts were automatically annotated based on accurate mass and isotope pattern matching within a 5 ppm tolerance. Dimeric adducts and multiply charged species are also included.

Adduct	$\Delta m/z$ (Da)	MeCN (Count)	FA (Count)
M+Na	+22.989218	712	726
M+K	+38.963158	429	445
M+MeCN+H	+42.033823	275	286
M+MeCN+Na	+64.022842	114	121
M+Na+H	+23.997037	179	180
M+FA+H	+46.005479	370	384
M+H-H_2O	–18.010565	381	401
M+2H	+2.014552 ($z = 2 \rightarrow m/z = (M + 2H)/2$)	164	163
2M+H	+M + 1.007276	58	57
2M+Na	+M + 22.989218	86	87
2M+K	+M + 38.963158	70	74

SI Table 2. Summary of common adducts detected in **RPLC-MS (negative ion mode)** following extraction with either MeCN alone or 1% formic acid (FA) followed by MeCN. The table lists theoretical mass shifts ($\Delta m/z$) for each adduct relative to the neutral monoisotopic mass (M), along with the number of features annotated using Progenesis QI. Adducts were identified using accurate mass and isotope pattern matching with a 5 ppm mass tolerance. Deprotonated species, water loss, chloride adducts, and dimeric ions are included.

Adduct	$\Delta m/z$ (Da)	MeCN (Count)	FA (Count)
M-H-H_2O	–19.017841	180	157
M+FA-H	+44.998203	223	212
M+Na-2H	+20.974666	304	288
M+K-2H	+36.948606	232	209
M+Cl	+34.969402	330	302
2M+FA-H	+M + 44.998203	21	31
2M-H	+M – 1.007276	27	25

SI Table S3. Summary of common adducts detected in **AEC-MS (negative ion mode)** following extraction with either MeCN alone or 1% formic acid (FA) followed by MeCN. The table lists theoretical mass shifts ($\Delta m/z$) for each adduct relative to the neutral monoisotopic mass (M), along with the number of features annotated using Progenesis QI. Adducts were identified using accurate mass and isotope pattern matching with a 5 ppm mass tolerance. Deprotonated species, water loss, chloride adducts, and dimeric ions are included.

Adduct	$\Delta m/z$ (Da)	MeCN (Count)	FA (Count)
M-H-H_2O	−19.017841	50	50
M+FA-H	+44.998203	27	25
M+Na-2H	+20.974666	14	17
M+K-2H	+36.948606	18	18
M+Cl	+34.969402	27	22
2M+FA-H	+M + 44.998203	3	3
2M-H	+M − 1.007276	31	29

Mechanistic insights: protein-metabolite interactions

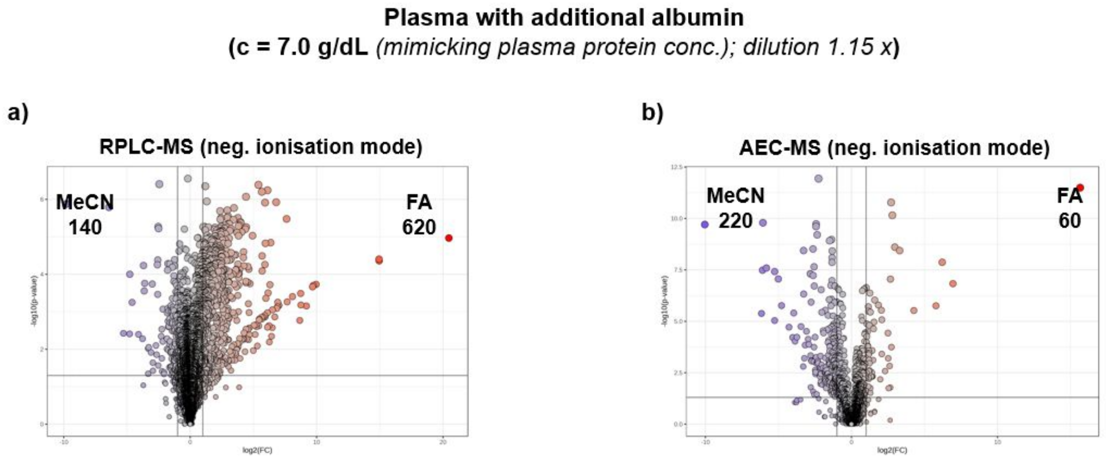

SI Figure 6. Effects of formic acid–protein interactions on metabolite extraction efficiency. Volcano plots showing differences in compound features between acidified and unacidified plasma samples with additional albumin (c = 7.0 g/dL) (t-test, $p < 0.05$). **(a)** RPLC-MS (neg. ionisation mode). **(b)** AEC-MS (neg. ionisation mode). Each extraction for repeated in five sample replicates.

Mechanistic insights: protein-metabolite interactions

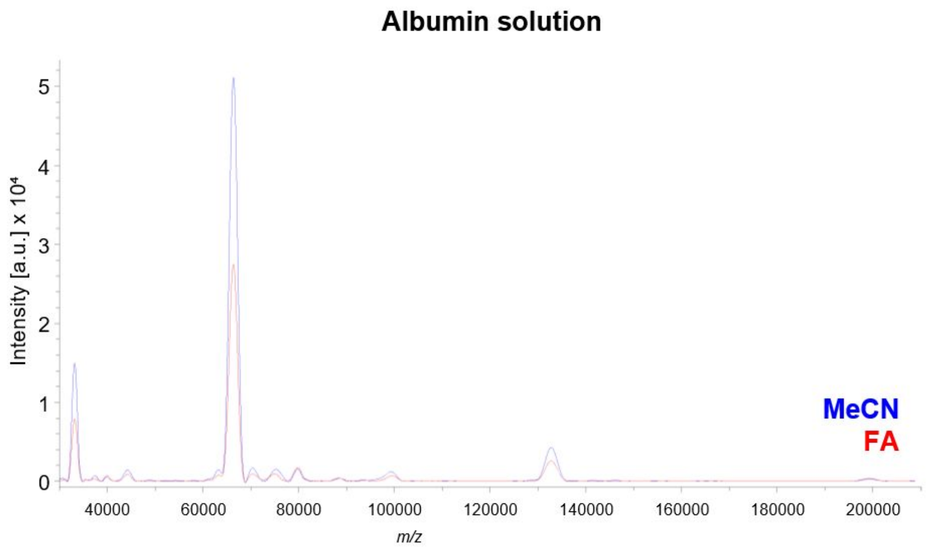

SI Figure 7. MALDI-MS spectra of the albumin protein pellet (aqueous bovine serum albumin (BSA); *c* = 7.0 g/dL) after extraction with MeCN (blue) and FA-MeCN (red). Spectra for the high mass range: 40-200kDa.

MALDI-MS Spectra for lower molecular masses (plasma)

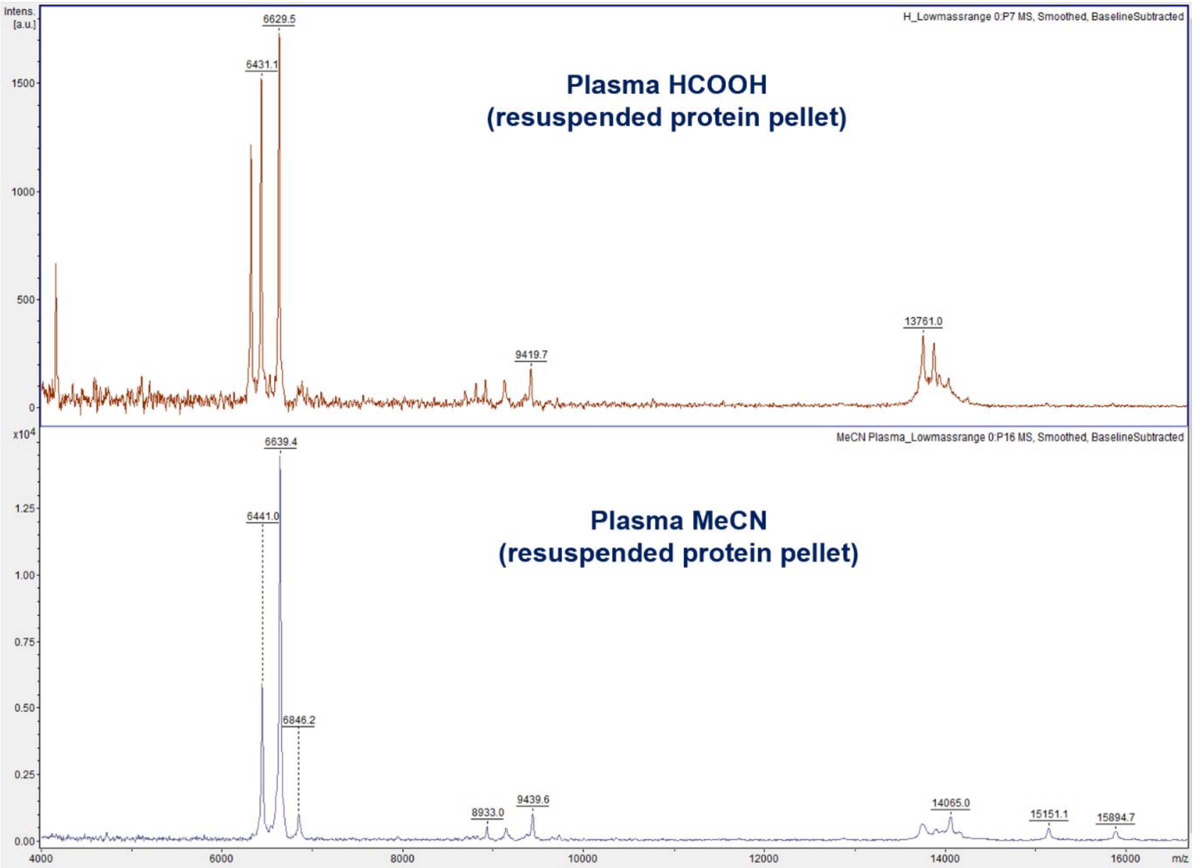

SI Figure 8. MALDI-MS spectra of the plasma protein pellet after extraction with MeCN (top) and FA-MeCN (bottom). Spectra for the low mass range: 4-16kDa.

MALDI-MS Spectra for lower molecular masses (albumin aqueous solution; c = 7.0 g/dL)

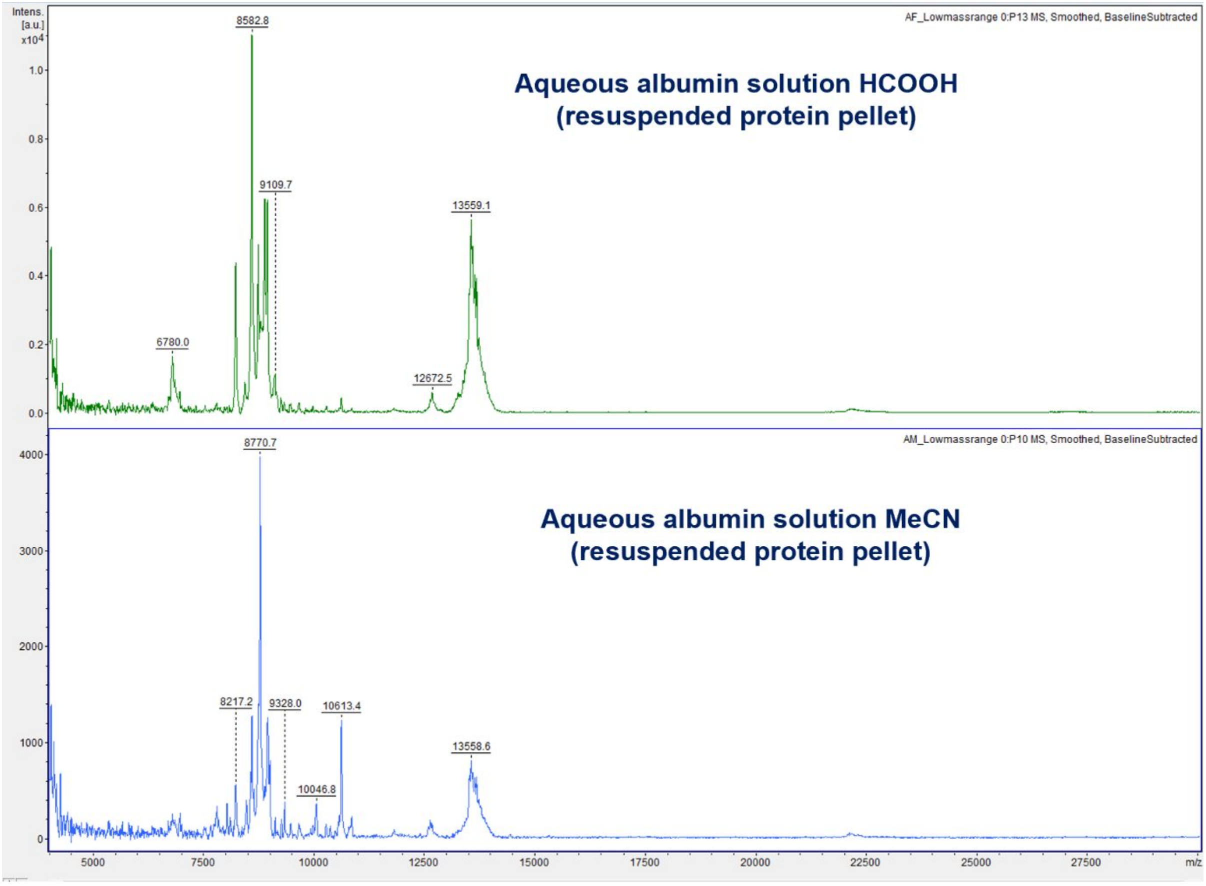

SI Figure 9. MALDI-MS spectra of the albumin protein pellet (aqueous bovine serum albumin (BSA); c = 7.0 g/dL) after extraction with MeCN (top) and FA-MeCN (bottom). Spectra for the low mass range: 4-16kDa.

Mechanistic insights: protein-metabolite interactions

SI Table 4. Compound-features with the lowest *p*-values in unpaired Student's t-tests comparing plasma extracts prepared with FA+MeCN versus MeCN alone (n = 5 replicates per group) in RPLC-MS positive ionisation mode. Positive t-statistic values indicate features more abundant in FA-treated samples. While the compound-features have not been definitively identified, their relatively high m/z values (approximately 280–780 *m/z*) and retention times (predominantly 10–12 minutes in an 18-minute gradient) suggest they are likely lipid-like metabolites, consistent with the enhanced recovery of hydrophobic compounds following disruption of protein–metabolite interactions by formic acid pre-treatment.

m/z	retention time (min)	t-statistic	*p*-value	FDR *p*-value
642.3703n	11.95	74.846	1.13E-12	6.53E-09
483.3255m/z	12.24	71.353	1.66E-12	6.53E-09
776.7881m/z	0.72	60.812	5.94E-12	1.56E-08
277.2128m/z	10.25	52.64	1.88E-11	3.71E-08
589.4303m/z	11.76	50.361	2.68E-11	3.84E-08
540.4470m/z	11.76	49.811	2.92E-11	3.84E-08
559.0890m/z	12.18	-47.559	4.23E-11	4.76E-08
588.2308n	9.44	44.415	7.29E-11	7.18E-08
406.3112n	11.42	41.912	1.16E-10	9.57E-08
545.4071m/z	12.51	41.662	1.21E-10	9.57E-08
635.3883m/z	11.95	40.968	1.39E-10	9.94E-08
689.0338m/z	12.22	-39.52	1.85E-10	1.17E-07
545.7428m/z	14.26	-39.034	2.04E-10	1.17E-07
486.8645m/z	13.42	-38.76	2.16E-10	1.17E-07
567.0929m/z	12.42	-38.613	2.22E-10	1.17E-07
565.0940m/z	12.2	-38.151	2.45E-10	1.21E-07
421.3404m/z	10.83	37.773	2.65E-10	1.23E-07
619.3092m/z	11.5	37.32	2.92E-10	1.27E-07
567.2511m/z	9.44	37.082	3.07E-10	1.27E-07
663.4587m/z	12.61	36.667	3.36E-10	1.32E-07
623.0654m/z	12.17	-35.948	3.93E-10	1.43E-07
459.3251m/z	12.46	35.863	4.00E-10	1.43E-07
657.1246m/z	12.63	-35.393	4.45E-10	1.52E-07
473.3456m/z	11.04	35.14	4.71E-10	1.55E-07
510.3564m/z	11.52	34.341	5.65E-10	1.78E-07
485.3357m/z	11.09	34.085	6.00E-10	1.81E-07
551.4270m/z	11.95	33.919	6.24E-10	1.81E-07
631.0758m/z	12.24	-33.741	6.50E-10	1.81E-07
508.3752m/z	11.73	33.639	6.66E-10	1.81E-07
625.4655m/z	12.51	33.509	6.87E-10	1.81E-07

Mechanistic insights: protein-metabolite interactions

SI Table 5. Comparison of proteinogenic amino acid levels in acetonitrile and formic acid–spiked acetonitrile extracts (HILIC-MS data). A total of 15 proteinogenic amino acids were detected, of which 4 showed significant differences (uncorrected *p*-values; no FDR adjustment to maximise sensitivity). All significantly altered amino acids were decreased in the acidified extracts, suggesting that the addition of formic acid does not promote cleavage of protein–peptide bonds to release free amino acids.

Amino acid	*p*-value
Alanine	0.911
Arginine	0.320
Glutamine	0.022
Glycine	0.021
Histidine	0.247
Isoleucine	0.147
Leucine	0.496
Lysine	0.157
Methionine	0.332
Phenylalanine	0.013
Proline	0.624
Serine	0.764
Tryptophan	0.012
Tyrosine	0.095
Valine	0.441

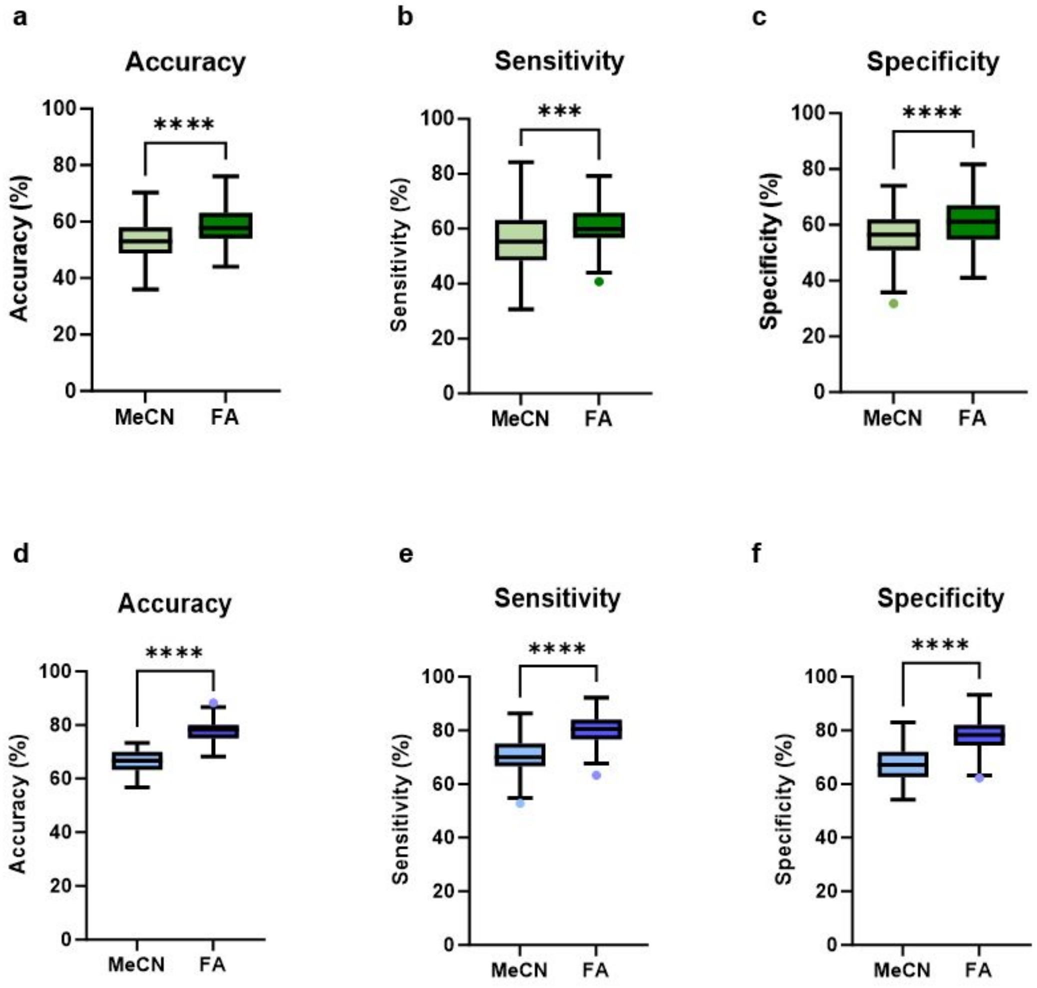

SI Figure 10. Formic acid–based extractions enhance the discriminatory capacity of supervised multivariate statistical models (orthogonal partial least squares discriminant analysis, OPLS-DA), as demonstrated in two independent cohorts: OPTIMA (early vascular dementia vs. early Alzheimer's disease) and VITACOG (vitamin B-treated patients vs. placebo controls). (a–c) Results for the OPTIMA cohort; (d–f) Results for the VITACOG cohort. Each panel compares accuracy, sensitivity, and specificity across 1000 diagnostic models constructed using either acetonitrile-based or formic acid–based extraction protocols.

- (a) OPTIMA accuracy ($p = 4.31\times10^{-6}$)
- (b) OPTIMA sensitivity ($p = 4.7\times10^{-4}$)
- (c) OPTIMA specificity ($p = 9.25\times10^{-6}$)
- (d) VITACOG accuracy ($p = 1.45\times10^{-52}$)
- (e) VITACOG sensitivity ($p = 4.18\times10^{-52}$)
- (f) VITACOG specificity ($p = 2.13\times10^{-27}$)

These results highlight the improved model performance achieved with formic acid–based extractions.

OPTIMA VIP scores

SI Table 6. List of the variables importance in projection identified from the AD vs VaD model (OPTIMA) for the MeCN-based extractions.

Rank	Compound-feature	Frequency	Average rank	Overall score
1	13.66_615.1469 m/z	533	8.6	62.3
2	2.80_114.0921 m/z	367	6.4	57.7
3	13.66_610.1929 m/z	470	10.7	43.9
4	8.30_244.2634 m/z	567	13.3	42.7
5	0.88_218.1398 m/z	650	17.5	37.2
6	7.08_255.5784 m/z	256	7.2	35.7
7	7.10_155.5415 m/z	288	8.1	35.6
8	8.56_153.0574 m/z	655	18.7	35.1
9	10.34_375.2855 m/z	618	18.0	34.4
10	0.79_132.0787 m/z	622	18.2	34.2
11	0.77_114.0678 m/z	599	18.2	32.8
12	0.77_114.0678 m/z	601	18.4	32.6
13	12.20_400.3476 m/z	592	19.6	30.2
14	0.79_136.0497 m/z	584	19.5	30.0
15	8.30_579.6032 m/z	281	10.0	28.1
16	7.92_370.0968 m/z	248	10.0	24.9
17	4.61_218.2116 m/z	183	7.7	23.8
18	5.97_212.0201 m/z	330	14.1	23.4
19	10.87_256.0872 m/z	204	8.8	23.2
20	0.88_231.0862 m/z	227	10.6	21.5
21	6.84_475.1081 m/z	201	9.5	21.1
22	8.35_597.4737 m/z	194	9.4	20.6
23	7.10_169.0647 m/z	218	10.9	20.0
24	7.75_477.1235 m/z	224	11.5	19.4
25	5.44_221.0128 m/z	160	8.3	19.2

SI Table 7. List of the variables importance in projection identified from the AD vs VaD model (OPTIMA cohort) for the FA-MeCN-based extractions.

Rank	Compound-feature	Frequency	Average rank	Overall score
1	9.12_316.3179 m/z	701	10.1	69.2
2	11.74_561.4805 m/z	362	6.0	60.6
3	11.50_281.2698 n	723	13.6	53.2
4	4.55_715.0295 m/z	446	9.8	45.6
5	4.55_856.8306 m/z	437	11.0	39.6
6	11.28_737.2232 m/z	334	9.2	36.3
7	7.49_249.1125 m/z	363	10.3	35.4
8	8.56_219.0681 m/z	289	8.6	33.7
9	12.46_662.6014 m/z	290	9.9	29.2
10	13.56_782.5610 m/z	600	20.8	28.8
11	13.51_806.5595 m/z	571	22.2	25.8
12	13.70_806.5598 m/z	570	22.3	25.5
13	11.21_521.3447 n	559	23.2	24.1
14	11.69_523.3588 n	562	23.6	23.8
15	11.02_495.3289 n	549	23.6	23.3
16	14.83_674.5643 m/z	551	23.7	23.2
17	4.68_269.9842 m/z	194	8.4	23.1
18	4.56_868.0168 m/z	298	13.2	22.6
19	10.32_270.6484 m/z	434	20.3	21.4
20	3.70_340.2595 m/z	227	10.8	21.1
21	4.68_237.9590 m/z	230	12.2	18.9
22	6.29_796.9330 m/z	227	12.1	18.8
23	2.80_114.0928 m/z	190	10.4	18.3
24	6.55_182.1191 m/z	311	18.8	16.6
25	6.31_797.2667 m/z	159	9.6	16.6

3.8 VITACOG VIP scores

SI Table 8. List of the variables importance in projection identified from the B vitamin-treated vs placebo model (VITACOG cohort) for the MeCN-based extractions.

Rank	Compound-feature	Frequency	Average rank	Overall score
1	11.90_514.3414 m/z	900	4.3	208.7
2	12.12_559.4056 m/z	460	3.3	138.6
3	10.51_271.1641 m/z	997	7.3	136.7
4	12.43_552.3962 m/z	397	3.3	122.0
5	10.71_417.7710 m/z	1000	9.8	102.0
6	9.89_293.2102 m/z	951	11.2	85.3
7	10.53_841.5444 m/z	366	5.0	73.2
8	11.47_839.5645 m/z	285	4.0	71.9
9	4.12_481.2622 m/z	662	9.4	70.2
10	10.54_885.5721 m/z	731	11.4	64.3
11	10.51_270.6550 m/z	738	12.2	60.3
12	11.01_519.8367 m/z	108	1.8	60.1
13	3.76_467.0084 m/z	284	5.0	56.7
14	5.90_236.1049 n	527	9.5	55.5
15	12.68_871.5632 m/z	112	2.1	53.6
16	0.65_127.0129 n	654	12.3	53.0
17	10.51_410.7730 m/z	129	2.7	48.1
18	18.00_123.0574 m/z	613	12.8	47.8
19	15.87_149.9562 m/z	112	2.4	45.8
20	3.28_484.5266 m/z	785	17.6	44.6
21	0.64_96.9622 m/z	981	22.4	43.8
22	8.66_250.1221 n	539	13.0	41.5
23	3.28_484.2760 m/z	757	18.7	40.5
24	11.42_783.9943 m/z	934	23.5	39.8
25	7.25_247.1219 m/z	753	19.2	39.2

SI Table 9. List of the variables importance in projection identified from the B vitamin-treated vs placebo model (VITACOG cohort) for the FA-MeCN-based extractions.

Rank	Compound-feature	Frequency	Average rank	Overall score
1	9.68_261.1482	1000	1.8	370.8
2	9.66_239.1683	310	1.1	287.6
3	7.18_837.6481	910	3.5	157.2
4	4.60_247.0146	1000	4.7	152.2
5	4.65_682.2554	935	5.6	139.6
6	7.18_838.2505	489	3.2	119.6
7	9.27_553.2105	228	1.7	110.4
8	7.18_838.4518	991	8.1	104.0
9	7.44_398.2472	995	10.4	95.9
10	9.53_410.2644	812	9.0	80.3
11	11.52_286.2388	182	2.4	76.3
12	7.75_166.0669 n	807	11.3	73.6
13	9.98_373.2453	959	13.4	64.5
14	10.87_796.9837	852	14.3	58.2
15	4.60_199.1359	978	17.0	56.7
16	4.65_685.2611	1000	18.7	49.6
17	0.73_218.9237	379	7.6	47.0
18	4.53_742.3158	67	1.3	43.7
19	7.44_376.2652	1000	22.3	42.4
20	4.44_472.2446	989	22.3	40.4
21	12.85_843.5655	626	14.3	38.5
22	8.97_365.1435	286	7.2	33.7
23	7.89_684.1699	581	15.6	31.8
24	12.96_603.5229	287	7.8	30.6
25	12.65_848.5702	944	27.7	30.2

SI Table 10. Comparison of compound-features extracted using FA-based and pure acetonitrile-based protocols. The compound-features were selected based on their associated VIP scores for the vitamin B-treated individuals (VB) vs placebo controls (P) model constructed using the FA-based extraction data. The table includes the median %CV (calculated from pooled QC samples), mean fold change (VB/P) for both extraction methods, and significance levels from unpaired two-sample t-tests comparing VB and P abundance (*p*-values: ***$p < 0.001$, **$p < 0.01$, *$p < 0.05$). Additionally, it reports the FA/MeCN mean abundance ratio, with associated t-test results (same p-value thresholds). Curation criteria for matching compound features between datasets included: retention time (Rt) error < 60 s, accurate mass error < 20 ppm, and isotope/fragmentation patterns match > 90%. Annotations: NA = compound-feature not detected; NA† = compound-feature not detected in any other analysed dataset; NA‡ = compound-feature not detected in acetonitrile-extracted clinical samples but detected in FA-based serum standards. For detailed t-test results, abundance and %CV data (for individual analytical subgroups), refer to SI Tables 7 and 8.

**FA Protocol**					**MeCN Protocol**	
Variable *Rt_m/z*	VIP Score	%CV	FC (VB/P)	Abundance ratio (FA/MeCN)	%CV	FC (VB/P)
9.68_261.1482	370.8	10.41	3.397***	0.79***	12.83	1.628***
9.66_239.1683	287.6	10.53	3.448***	0.74***	13.59	1.630***
7.18_837.6481	157.2	14.64	2.005*	NA†	NA†	NA†
4.60_247.0146	152.2	12.65	0.521*	NA‡	NA‡	NA‡
4.65_682.2554	139.6	16.22	1.444**	0.72***	7.70	1.262^{ns}
7.18_838.2505	119.6	12.77	0.255***	3.02***	11.23	0.819^{ns}
9.27_553.2105	110.4	13.65	0.599*	NA‡	NA‡	NA‡
7.18_838.4518	104.0	13.81	0.284***	1.93***	11.20	0.777**
7.44_398.2472	95.9	10.74	2.863***	0.80**	7.90	1.627***
9.53_410.2644	80.3	10.28	3.091***	1.04***	10.72	1.586***
11.52_286.2388	76.3	10.57	1.961***	4.58***	17.48	0.911^{ns}
7.75_166.0669 n	73.6	21.37	0.567***	0.46***	6.74	0.775**
9.98_373.2453	64.5	14.71	0.514***	NA‡	NA‡	NA‡
10.87_796.9837	58.2	10.34	0.695**	0.33***	5.73	0.715***
4.60_199.1359	56.7	10.73	0.843^{ns}	0.86^{ns}	9.29	1.551**
4.65_685.2611	49.6	21.07	0.336*	1.96***	24.52	0.922^{ns}
0.73_218.9237	47.0	17.46	0.848**	0.08***	6.46	0.675***
4.53_742.3158	43.7	10.36	0.818^{ns}	0.13***	6.34	0.674^{ns}
7.44_376.2652	42.4	19.67	5.967***	0.59***	9.12	1.963***
4.44_472.2446	40.4	16.33	0.756*	0.28***	4.14	1.003^{ns}
12.85_843.5655	38.5	10.37	0.893*	0.30***	11.10	0.777***
8.97_365.1435	33.7	16.74	1.749***	5.18***	17.97	0.524***
7.89_684.1699	31.8	15.65	2.143^{ns}	NA‡	NA‡	NA‡
12.96_603.5229	30.6	14.61	0.837**	0.98*	15.31	0.616***
12.65_848.5702	30.2	10.65	1.008^{ns}	1.92***	9.60	0.634^{ns}

We compared the top 20 compound-features (by VIP score) detected in both FA- and MeCN-extracted datasets. No consistent pattern was observed in their abundance or %CV, with FA treatment leading to both increases and decreases depending on the compound-feature. The average %CV in QC samples was significantly lower in MeCN extracts ($p = 0.026$), and 12 of the 20 features showed higher abundance in MeCN. However, OPLS-DA separation is likely driven more by relative differences and reduced background noise than absolute abundance. Fold-changes between treated and placebo groups were enhanced in FA-treated samples (Table 6, SI Table 8), suggesting improved capture of biologically relevant variation through this extraction protocol (FC $p = 0.007$ (for FC < 1; the reversed value (1/FC) was used for *p* value calculations).

SI Table 11. Compound-features with the highest VIP scores in the comparison between the vitamin B-treated (VB) and placebo (P) groups, based on FA-extracted data. The table includes the coefficient of variance (%CV) for the pooled QC samples, as well as for two sets of analytical samples: one set consisting only of vitamin B-treated individuals and another consisting only of placebo controls. The %CV values are provided for both FA-based and pure acetonitrile extractions. Curation criteria for matching compound features between datasets included: retention time (Rt) error < 60 s, accurate mass error < 20 ppm, and isotope/fragmentation patterns match > 90%. Annotations: NA = feature not detected; NA† = feature not detected in any other analysed dataset; NA‡ = feature not detected in acetonitrile-extracted clinical samples but detected in FA-based serum standards.

Variable *Rt_m/z*	FA			MeCN		
	%CV QC	%CV VB	%CV Placebo	%CV QC	%CV VB	%CV Placebo
9.68_261.1482	10.41	59.25	124.13	12.83	83.89	32.61
9.66_239.1683	10.53	52.33	103.73	13.59	79.75	49.98
7.18_837.6481	14.64	50.01	68.37	NA†	NA†	NA†
4.60_247.0146	12.65	109.35	92.42	NA‡	NA‡	NA‡
4.65_682.2554	16.22	211.9	181.88	7.70	63.78	48.37
7.18_838.2505	12.77	127.21	178.12	11.23	60.40	57.13
9.27_553.2105	13.65	74.11	81.11	NA‡	NA‡	NA‡
7.18_838.4518	13.81	133.01	175.72	11.20	55.91	48.14
7.44_398.2472	10.74	60.03	52.2	7.90	53.55	57.17
9.53_410.2644	10.28	82.07	79.48	10.72	40.94	34.92
11.52_286.2388	10.57	33.99	66.49	17.48	189.17	55.95
7.75_166.0669 n	21.37	85.93	57.12	6.74	73.69	57.33
9.98_373.2453	14.71	73.47	50.16	NA‡	NA‡	NA‡
10.87_796.9837	10.34	122.27	73.04	5.73	88.92	34.27
4.60_199.1359	10.73	46.31	81.64	9.29	32.57	80.21
4.65_685.2611	21.07	103.2	90.19	24.52	67.35	50.14
0.73_218.9237	17.46	131.2	137.48	6.46	14.82	20.82
4.53_742.3158	10.36	75.65	64.4	6.34	82.733	77.91
7.44_376.2652	19.67	126.68	125.36	9.12	47.95	67.18
4.44_472.2446	16.33	60.64	57.51	4.14	71.34	46.17
12.85_843.5655	10.37	52.71	32.76	11.1	38.56	44.30
8.97_365.1435	16.74	55.21	169.08	17.97	237.17	260.03
7.89_684.1699	15.65	75.23	104.52	NA‡	NA‡	NA‡
12.96_603.5229	14.61	49.59	25.28	15.31	27.00	33.82
12.65_848.5702	10.65	44.52	26.05	9.60	79.72	81.18

SI Table 12. Compound-features with the highest VIP scores in the comparison between the vitamin B-treated (VB) and placebo (P) groups, based on FA-extracted data. The table includes the mean fold change in compound features for VB vs P individuals in both acetonitrile and FA-based extracts. The corresponding *p*-values, based on a t-test comparing the mean abundances for each condition, are also provided. Additionally, the ratio of compound-feature abundance between acetonitrile and FA-based extracts is shown, along with p-values (from t-tests) for these comparisons. Curation criteria for matching compound features between datasets included: retention time (Rt) error < 60 s, accurate mass error < 20 ppm, and isotope/fragmentation patterns match > 90%. Annotations: NA = feature not detected; NA† = feature not detected in any other analysed dataset; NA‡ = feature not detected in acetonitrile-extracted clinical samples but detected in FA-based serum standards.

Variable *Rt_m/z*	FA		MeCN		QC FA vs MeCN	
	FC (VB/P)	*p*-value abundance	FC (VB/P)	*p*-value abundance	QC Abundance	*p*-value
9.68_261.1482	3.397	2.76E-06	1.628	1.64E-07	0.79	1.83E-07
9.66_239.1683	3.448	1.55E-06	1.630	1.99E-05	0.74	6.30E-13
7.18_837.6481	2.005	0.0415	NA†	NA†	NA†	NA†
4.60_247.0146	0.521	0.0131	NA‡	NA‡	NA‡	NA‡
4.65_682.2554	1.444	0.0070	1.262	0.6885	0.72	3.26E-18
7.18_838.2505	0.255	6.09E-05	0.819	0.0554	3.02	2.88E-14
9.27_553.2105	0.599	0.0281	NA‡	NA‡	NA‡	NA‡
7.18_838.4518	0.284	2.45E-04	0.777	0.0046	1.93	5.23E-12
7.44_398.2472	2.863	3.67E-07	1.627	5.38E-10	0.80	0.0070
9.53_410.2644	3.091	5.73E-08	1.586	1.33E-10	1.04	0.29840
11.52_286.2388	1.961	1.18E-04	0.911	0.0803	4.58	1.76E-11
7.75_166.0669 n	0.567	6.64E-04	0.775	0.0038	0.46	3.03E-21
9.98_373.2453	0.514	2.97E-09	NA‡	NA‡	NA‡	NA‡
10.87_796.9837	0.695	0.0016	0.715	1.51E-09	0.33	1.33E-20
4.60_199.1359	0.843	0.1087	1.512	0.0018	0.86	0.3965
4.65_685.2611	0.336	0.0216	0.922	0.6148	1.96	7.59E-23
0.73_218.9237	0.848	0.0063	0.675	6.24E-07	0.08	4.73E-10
4.53_742.3158	0.818	0.1036	0.674	0.1814	0.13	1.28E-20
7.44_376.2652	5.967	3.04E-04	1.963	1.37E-11	0.59	1.80E-08
4.44_472.2446	0.756	0.0399	1.003	0.7448	0.28	1.92E-14
12.85_843.5655	0.893	0.0414	0.777	4.54E-06	0.30	1.66E-11
8.97_365.1435	1.749	3.72E-04	0.524	0.0008	5.18	3.83E-10
7.89_684.1699	2.143	0.5635	NA‡	NA‡	NA‡	NA‡
12.96_603.5229	0.837	0.0067	0.616	1.16E-05	0.98	0.0260
12.65_848.5702	1.008	0.7637	0.634	0.3945	1.92	2.60E-19
